# The predictive value of BOAH scale for screening obstructive sleep apnea in patients at a sleep clinic in Scotland

**DOI:** 10.1007/s11325-020-02114-0

**Published:** 2020-06-10

**Authors:** Agata Gabryelska, Łukasz Mokros, Grzegorz Kardas, Michał Panek, Renata Riha, Piotr Białasiewicz

**Affiliations:** 1 Department of Sleep Medicine and Metabolic Disorders, Medical University of Lodz, Mazowiecka 6/8, 92-215 Lodz, Poland; 2 Department of Sleep Medicine, Royal Infirmary Edinburgh, Edinburgh, UK; 3 Department of Clinical Pharmacology, Medical University of Lodz, Lodz, Poland; 4 Department of Internal Medicine, Asthma and Allergy, Medical University of Lodz, Lodz, Poland

**Keywords:** OSA, Predictive value, BOAH, PSG

## Abstract

**Objectives:**

The study aimed to evaluate the diagnostic value of an original questionnaire for obstructive sleep apnea (OSA), the BOAH scale, and its ability to prioritize patients at high risk for OSA for polysomnography (PSG) examination.

**Methods:**

The analysis included 273 patients referred to the Department of Sleep Medicine of the Royal Infirmary, Edinburgh, Scotland. The BOAH scale is comprised of 5 parameters: BMI (≥ 30 kg/m^2^ gives 1 point, ≥ 35 kg/m^2^ 2 points), presence of witnessed apneas during sleep (1 point), patient age ≥ 50 years (1 point), and history of hypertension (1 point). Patients were divided into three study groups depending on OSA severity defined by the apnea-hypopnea index (AHI): at least mild (AHI ≥ 5), at least moderate (AHI ≥ 15), and severe (AHI ≥ 30) OSA based on polysomnography examination.

**Results:**

In the group of patients with severe OSA, the best BOAH cutoff point was 4 points based upon the Youden index. With 4 points, the area under the receiver operating characteristic (ROC) curve was 0.778 (95% CI 0.721–0.834). Sensitivity and specificity were 57% and 89%, respectively, yielding a positive and negative predictive value of 75% and 78%, respectively, for diagnosis of severe OSAS in a patient sample with a pre-test probability for severe OSA at 37%.

**Conclusions:**

The BOAH scale in this group of Scottish patients performed comparably to other available questionnaires and scales while being shorter and simpler. The findings suggest that the BOAH scale should be considered as a useful instrument in OSA diagnosis and prioritization of high-risk patients for PSG examination.

## Introduction

Obstructive sleep apnea (OSA) is a prevalent sleep disorder characterized by recurrent pauses in breathing during sleep caused by the collapse of upper airways. It is associated with metabolic and cardiovascular comorbidities [1]. The primary risk factor for OSA is obesity, which leads to an increased volume of pharyngeal soft tissue. It is estimated that 90% of patients suffering from OSA are overweight and over 60% is obese [2]. The important unmodifiable risk factors for OSA are age over 50 years and male sex [3]. The prevalence of the disorder in the general adult population currently ranges between 9 to 38% [4]. However, some studies estimate that at least a moderate form of OSA may affect up to 23% women and 49% of men [5].

The polysomnography (PSG) is considered as a gold standard in OSA diagnosis. Unfortunately, access to this diagnostic procedure is limited due to its cost and the low number of specialized diagnostic sleep clinics. Thus, there is a great need to develop a simple and effective questionnaire to assess the probability of OSA and prioritize patients at high risk for the diagnostic PSG. One of the most commonly used tools is the STOP-BANG questionnaire. It consists of four subjective parameters (STOP: snoring, tiredness, observed apnea, and history of arterial hypertension) and four demographic items (BANG: BMI, age, neck circumference, and gender) [6].

Other screening tests, including the Berlin questionnaire, STOP, Epworth sleepiness scale, and recently created NoSAS scale, are all used to identify high-risk patients [7, 8]. However, some of these tools are lengthy and complicated or require an upper airway assessment, making them inconvenient to use. Meta-analyses of different tools showed that the STOP-BANG questionnaire is the most accurate in OSA diagnosis [7]. However, recent studies suggest that NoSAS is a superior screening tool to detect clinically significant sleep-disordered breathing.

BOAH scale is a shortened version of the STOP-BANG questionnaire that consists of variables that can easily be assessed by a physician: BMI, a history of witnessed apneas during sleep, a patient’s age, and a history of hypertension [9, 10]. Originally, the scale was created in Sleep and Respiratory Disorders Centre (Lodz, Poland), specializing in OSA diagnostics [9]. Therefore, the aim of this study was to assess the diagnostic value of this scale among patients referred to a sleep clinic due to diverse sleep disorders and examine its ability to prioritize patients with a high risk of OSA for PSG examination.

## Materials and methods

The retrospective study involved 273 consecutive patients referred to the Department of Sleep Medicine of the Royal Infirmary (Edinburgh, Scotland) due to presumptive sleep disorder diagnosis between June 2015 and July 2016, who underwent diagnostic PSG examination. Based on data from patients’ history and examination, a BOAH scale score was calculated based on the following scoring criteria: BMI (≥ 35 kg/m^2^–2 points, ≥ 30 kg/m^2^–1point), witnessed apneas during sleep (1 point), patient’s age (≥ 50–1 point) and a history of arterial hypertension (1 point).

### Polysomnography

After the admission to the sleep laboratory (21:00 h ± 0.5 h) patients underwent body mass, height, heart rate, and blood pressure measurements. Following channels were used to record standard PSG: electroencephalography (C4\A1, C3\A2), chin muscles and anterior tibialis electromyography, electrooculography, measurements of oronasal airflow (a thermistor and cannula), snoring, body position, respiratory movements of chest and abdomen (plethysmographic belts), unipolar electrocardiogram, and hemoglobin oxygen saturation (SaO_2_). Sleep stages were scored according to the criteria based on a 30 s epoch standard. Apnea was defined as a reduction of airflow to less than 10% of the baseline for at least 10 s while hypopnea as at least 30% reduction of airflow for at least 10 s, co-occurring with decrease in SaO_2_ over 3% or an arousal. Electroencephalogram arousals were scored according to the American Academy of Sleep Medicine guidelines [11]. Additionally, PSG was extended by video recording of the patient while asleep.

All patients gave written informed consent for diagnostic polysomnography. The study was conducted in accordance with the amended Declaration of Helsinki.

### Statistical analysis

The data were analyzed with the Statistica 13.1 software (StatSoft, Tulsa, USA). Data distribution was tested with the Shapiro-Wilk test. The Student’s *t* test or Mann Whitney *U* was used to compare continuous variables in case of normal and non-normal distribution of data, respectively. The frequencies were compared with Chi^2^ test. Receiver operating characteristic (ROC) curves were created, and area under the curve (AUC) was calculated for AHI ≥ 5, ≥ 15, and ≥ 30 events/h using BOAH score as a predictor variable. Based on Youden index, cutoff points for the scale score were chosen. A value of *p* < 0.05 was considered significant.

## Results

Characteristics of the study group, including BOAH scores, are shown in Table 1.

**Table 1:** Characteristics of the study groups

	Study group	No OSA (AHI < 5)	At least mild OSA (5 ≤ AHI)	At least moderate OSA (15 ≤ AHI)	Severe OSA (30 ≤ AHI)
*N*	273	33	240	175	102
Age (years)	49.4 ± 17.3	41.4 ± 13.9	51.0± 13.3	52.0 ± 12.3	54.9 ± 12.2
BMI (kg/m^2^)	30.5 (26.7–36.1)	26.4 (23.7–31.7)	30.9 (27.2–36.3)	32.0 (28.3–37.5)	33.1 (29.1–38.7)
AHI	21.7 (9.2–41.2)	2.6 (1.8–3.5)	25.6 (14.0–45.6)	33.4 (23.8–61.2)	52.6 (36.9–75.0)
Gender	143 M (52%)	8 M (24%)	135 M (56%)	104 M (59%)	58 M (57%)
Observed apneas	171 (62%)	9 (27%)	162 (68%)	130 (74%)	80 (78%)
Arterial hypertension	110 (40%)	7 (21%)	103 (43%)	91 (52%)	70 (69%)
BOAH score	2 (1–4)	1 (0–2)	3 (1–4)	3 (2–4)	4 (2–4)

Data are shown as mean ± SD or median (IQR)

*AHI* apnea-hypopnea index, *BMI* body mass index, *M* male, *OSA* obstructive sleep apnea

The initial risk for at least mild OSA was 88%, while for the severe 37%. Sensitivity, specificity, positive predictive value (PPV), negative predictive value (NPV), and number of individuals for each BOAH score are showed in Table 2. BOAH score of 4 had PPV of 97% for at least mild and 88% for at least moderate form of OSA. Furthermore, the BOAH score of less than 2 had NPV of 97% for severe and 84% for at least moderate OSA, while score of less than 3 had NPV of 85% for severe OSA.

**Table 2:** Predictive values for all BOAH scores for OSA diagnosis

OSA severity	Initial risk	BOAH score	*n*	Sensitivity	Specificity	PPV	NPV
At least mild (AHI ≥ 5)	88%	0	25	–	–	–	–
		1	44	89%	39%	91%	34%
		2	47	71%	70%	94%	25%
		3	49	51%	82%	95%	19%
		4	56	31%	94%	97%	16%
		5	19	8%	97%	95%	13%
At least moderate (AHI ≥ 15)	64%	0	6	–	–	–	–
		1	29	97%	33%	72%	84%
		2	33	80%	58%	77%	62%
		3	39	61%	77%	82%	52%
		4	51	39%	91%	88%	46%
		5	17	9%	97%	84%	38%
Severe (AHI ≥ 30)	37%	0	1	–	–	–	–
		1	13	99%	22%	42%	97%
		2	15	86%	46%	48%	85%
		3	15	71%	67%	55%	80%
		4	43	57%	89%	75%	78%
		5	15	14%	97%	74%	66%

*AHI* apnea-hypopnea index, *NPV* negative predictive value, *OSA* obstructive sleep apnea, *PPV* positive predictive value

**Fig. 1:**
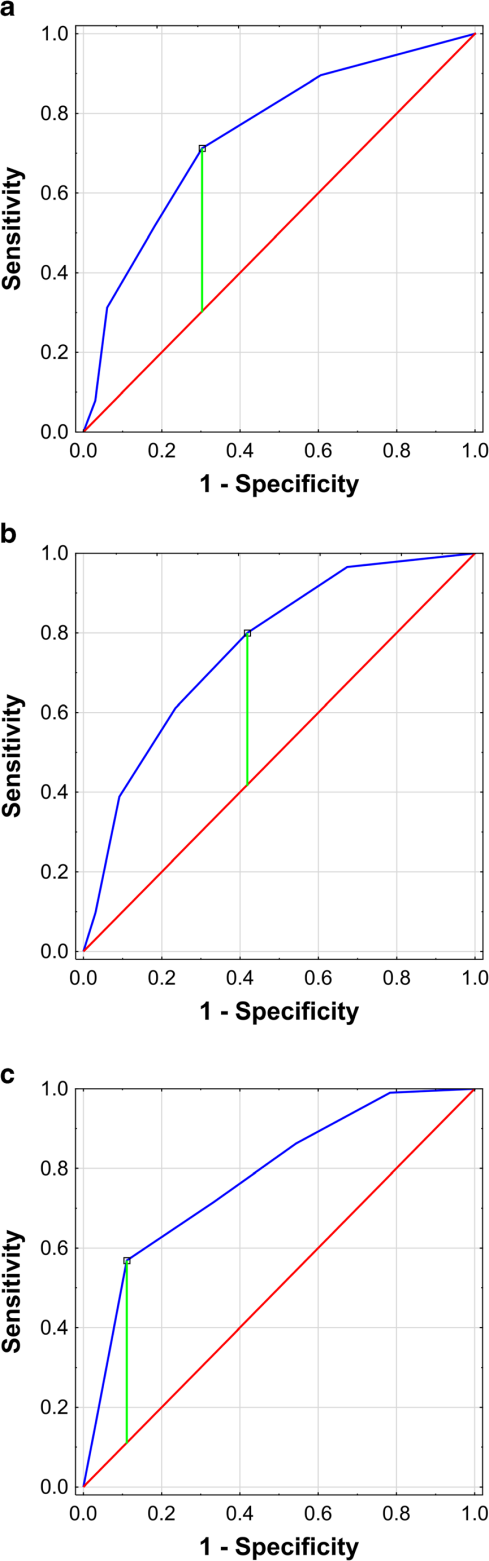
ROC curves for BOAH scale in mild, moderate, and severe OSA. Receiver operating characteristic (ROC) curves for BOAH scale for: **a**—mild obstructive sleep apnea (OSA), **b**—moderate OSA, **c**—severe OSA

Based on Youden index, cutoff level of 4 points in BOAH scale was chosen for severe OSA, which resulted in sensitivity of 57%, specificity of 89%, positive predictive value (PPV) of 75%, and negative predictive value (NPV) of 78%. The AUC was the largest for severe OSA − 0.776 (95% CI 0.718–0.833) (*p* < 0.001). The ROC curves for BOAH scale in mild, moderate, and severe OSA are shown in Table 3 and Fig. 1.

**Table 3:** ROC curve attributes for BOAH scale in at least mild, moderate, and severe OSA groups

OSA severity	Pre-test probability	Cutoff point	Youden index	Sensitivity	Specificity	PPV	NPV	AUC	95% CI	*p*
At least mild (AHI ≥ 5)	88%	2	0.41	71%	70%	94%	25%	0.749	0.660–0.838	< 0.001
At least moderate (AHI ≥ 15)	64%	2	0.38	80%	58%	77%	62%	0.764	0.704–0.823	< 0.001
Severe (AHI ≥ 30)	37%	4	0.46	57%	89%	75%	78%	0.778	0.721–0.834	< 0.001

*AHI* apnea-hypopnea index, *AUC* area under curve, *CI* confidence interval, *NPV* negative predictive value, *OSA* obstructive sleep apnea, *PPV* positive predictive value, *ROC* receiver operating curve

## Discussion

With a continuing increase in OSA prevalence and limited access to PSG examination it is important to prioritize patients with a higher risk for the severe form of the disorder. Multiple scales and questionnaires have been created to screen patients for OSA and refer them for PSG examination. Two of the most popular tools include the STOP-BANG questionnaire [12] and the recently developed NoSAS score [8]. However, both scales include either numerous parameters, 8 in the STOP-BANG questionnaire and multiple scoring levels for parameters in NoSAS. Newly created BOAH scale is one of the simplest tools available with only 4 variables and only BMI with 2 different levels of scoring. Additionally, 3 remaining variables: observed apneas, age over 50, and history of hypertension are collectible at history taking, allowing for the calculation of BOAH score in a short time, making the tool more practical and convenient.

BOAH scale had greater diagnostic value compared to the STOP-BANG questionnaire in the meta-analysis, which at the optimal cutoff point of 5 for severe OSA had both specificity and sensitivity of 66% [13]. This study is the second one to investigate the usefulness of the BOAH scale among sleep clinic patients. Previously, it was studied among 1135 patients, who underwent PSG examination in Sleep and Respiratory Disorders Centre (Lodz, Poland). In that study, the BOAH scale presented greater predictive values than the STOP-BANG questionnaire at the optimal cut-off points [9]. As the aforementioned center deals exclusively with OSA patients, in the present study, the scale was evaluated in a center attending to various sleep disorders to verify its potential diagnostic value on a more heterogeneous patient group. The BOAH scale disclosed the highest AUC (0.78) for severe OSA, at a cutoff level of 4, high specificity (89%), PPV (75%), and NPV (78%). The BOAH scale has had similar AUC results to NoSAS for clinically significant OSA (compared to both: the original and validating cohort, 0.74 and 0.81, respectively) [8], while having simpler scoring criteria suggesting it can be used as a robust tool for prioritizing patients with a high risk of severe OSA, for PSG examination. Furthermore, STOP-BANG meta-analysis [13] shows that PPV of 97% for mild and 88% for moderate OSA is achieved for a score of 7 in this questionnaire, which directly corresponds to the predictive values obtained for the BOAH score of 4. This shows that BOAH scale has the same diagnostic values as STOP in mild and moderate OSA diagnoses. Additionally, with 99% sensitivity, BOAH score of 1 has 97% NPV for severe OSA with only one false negative, allowing for quick prioritization of patients for PSG examination, while the STOP-BANG for the same severity and sensitivity has NPV of 89% at the cutoff point of 2. The primary limitation of the study is the lack of direct comparison between STOP-BANG and BOAH scores in the study group. Unfortunately, less than 10% of individuals included in the analysis had information regarding their neck circumference, which is mandatory for STOP-BANG. This shows that a limited number of parameters in the scale are advantageous as it is more likely that necessary data will be collected. In this manner, it may be more friendly to use GPs assessing OSA risk before referral. Yet, in general, neck circumference is recorded for most patients while they are admitted to the sleep clinic, which might make this missing data a negligible problem. The relatively small size of the study group could be also considered as a limitation to the study. Nevertheless, obtained results for the BOAH scale were comparable to the original study on a larger group of patients [9], which suggests that the examined group was representative.

BOAH scale is a valuable tool in OSA diagnosis and assessment of the risk of the disorder. It offers similar predictive values to other available tools while being shorter and easier in use. Therefore, it should be considered as a useful tool in clinical practice.

## References

[1] Gabryelska A, Łukasik ZM, Makowska JS, Białasiewicz P (2018). Obstructive sleep apnea: from intermittent hypoxia to cardiovascular complications via blood platelets. Front Neurol.

[2] Mokros Ł, Kuczynski W, Gabryelska A, Franczak Ł, Spałka J, Białasiewicz P (2018). High negative predictive value of normal body mass index for obstructive sleep apnea in the lateral sleeping position. J Clin Sleep Med.

[3] Punjabi NM (2008). The epidemiology of adult obstructive sleep apnea. Proc Am Thorac Soc.

[4] Senaratna CV, Perret JL, Lodge CJ, Lowe AJ, Campbell BE, Matheson MC, Hamilton GS, Dharmage SC (2017). Prevalence of obstructive sleep apnea in the general population: a systematic review. Sleep Med Rev.

[5] Heinzer R, Vat S, Marques-Vidal P, Marti-Soler H, Andries D, Tobback N, Mooser V, Preisig M, Malhotra A, Waeber G, Vollenweider P, Tafti M, Haba-Rubio J (2015). Prevalence of sleep-disordered breathing in the general population: the HypnoLaus study. Lancet Respir Med.

[6] Chung F, Yegneswaran B, Liao P, Chung SA, Vairavanathan S, Islam S, Khajehdehi A, Shapiro CM (2008). STOP questionnaire: a tool to screen patients for obstructive sleep apnea. Anesthesiology.

[7] Chiu H-Y, Chen P-Y, Chuang L-P, Chen NH, Tu YK, Hsieh YJ, Wang YC, Guilleminault C (2017). Diagnostic accuracy of the Berlin questionnaire, STOP-BANG, STOP, and Epworth sleepiness scale in detecting obstructive sleep apnea: a bivariate meta-analysis. Sleep Med Rev.

[8] Marti-Soler H, Hirotsu C, Marques-Vidal P, Vollenweider P, Waeber G, Preisig M, Tafti M, Tufik SB, Bittencourt L, Tufik S, Haba-Rubio J, Heinzer R (2016). The NoSAS score for screening of sleep-disordered breathing: a derivation and validation study. Lancet Respir Med.

[9] Mokros L, Kuczynski W, Gabryelska A, Bialasiewicz P (2016) BOAH–a shorter version of STOP-BANG questionnaire for prioritizing patients for polysomnography in esleep clinic. ERS international congress 2016 abstracts PA2310. 10.1183/13993003.congress-2016.PA2310

[10] Gabryelska A, Mokros Ł, Riha R et al (2018) The predictive value of BOAH scale among patients of sleep disorders clinic at the Department of Sleep Medicine in Edinburgh. Porto Biomed. J. YES Meeting (62)

[11] Kapur VK, Auckley DH, Chowdhuri S, Kuhlmann DC, Mehra R, Ramar K, Harrod CG (2017). Clinical practice guideline for diagnostic testing for adult obstructive sleep apnea: an American Academy of Sleep Medicine Clinical Practice Guideline. JCSM.

[12] Chung F, Abdullah HR, Liao P (2016). STOP-Bang questionnaire: a practical approach to screen for obstructive sleep apnea. Chest.

[13] Nagappa M, Liao P, Wong J, Auckley D, Ramachandran SK, Memtsoudis S, Mokhlesi B, Chung F (2015). Validation of the STOP-Bang questionnaire as a screening tool for obstructive sleep apnea among different populations: a systematic review and meta-analysis. PLoS One.

